# A Giant Simple Liver Cyst That Caused Increases in Serum CA 19-9 and CA 15-3 Levels

**DOI:** 10.14740/jocmr1950e

**Published:** 2014-09-09

**Authors:** Bulent Dinc, Ayhan Mesci, Selcan Enver Dinc, Alten Oskay

**Affiliations:** a Department of Surgery, Ataturk State Hospital, Antalya, Turkey; b Department of Surgery, Akdeniz University Faculty of Medicine, Antalya, Turkey; c Department of Emergency, Isparta State Hospital, Isparta, Turkey; d Department of Emergency, Denizli State Hospital, Denizli, Turkey

**Keywords:** Liver, Cysts, CA 19-9, CA 15-3

## Abstract

Simple cysts (SCs) of the liver are not associated with the biliary malformations in intrahepatic bile duct biliary. Seen in 0.1% to 7% of adult population, biliary malformations are more common in women. The levels of glycoprotein-like tumor markers (carbohydrate antigen (CA) 19-9) in the cysts and serum could be high. Although studies regarding CA 19-9 exist, sufficient data on cancer antigen (CA) 15-3 are not available. This case is about a 76-year-old woman who complained of painless intra-abdominal mass. The patient with a giant simple cyst extending from the gallbladder to the pelvis had preoparative CA 19-9 and CA 15-3 serum levels of 87.3 IU/L and 37 IU/L respectively. It was observed that CA 19-9 levels had decreased to 36 IU/L and CA 15-3 to 28.1 IU/L in blood samples taken in the third month after the surgery. There is a need for comprehensive studies to investigate the relationship between the size of the cyst and biomarkers (including markers such as CA 15-3) in the assesment of liver SC.

## Introduction

Liver cysts belong to a heterogeneous group of diseases. They are classified as congenital, traumatic, neoplastic, or parasitic. The congenital type, being one of the most important group, includes simple cysts (SCs) and polycystic liver diseases [1].

SCs of the liver are biliary malformations that are not associated with the intrahepatic bile duct. Seen in 0.1% to 7% of adult population, biliary malformations are more common in women. The cysts are usually asymptomatic and are smaller than 3 cm [1]. Liver functions of the patients with cysts are usually within normal limits. The level of glycoprotein-like tumor markers (carbohydrate antigen (CA) 19-9, carcinoembryonic antigen (CEA)) in the cysts and the serum could be high [2]. Although studies regarding the CA 19-9 exist, sufficient data on cancer antigen (CA) 15-3 are not available.

A giant simple liver cyst that caused an increase in serum CA 19-9 and CA 15-3 levels is presented here.

## Case Report

**Figure 1:**
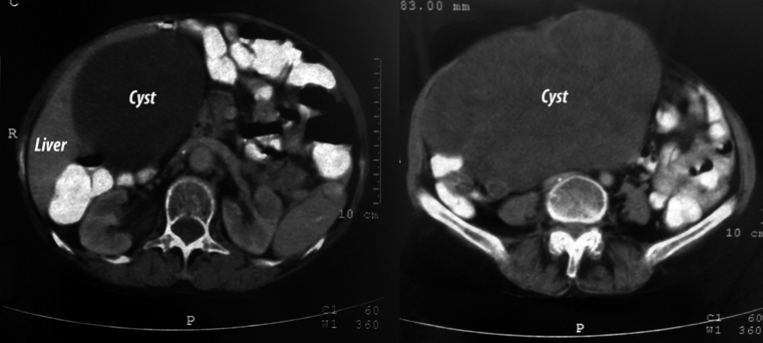
Computed tomography image of the giant cyst.

A 76-year-old woman who had complains of painless intra-abdominal mass was admitted to the clinic. During physical examination, an elastic mass extending from the right upper quadrant to the pelvis was detected. Laboratory tests showed normal liver functions. Serum CA 19-9 level of 87.3 IU/L (reference: < 37 IU/L), CA 15-3 level of 37 IU/L (reference: < 32.4 IU/L) and CEA level of 4.04 ng/mL (reference: 0 - 5 ng/mL) were detected. The existence of a cystic mass measured 21 × 11 cm extending from the gallbladder to the pelvic was detected with abdominal ultrasonography (USG) and computed tomography (CT) (Fig. 1).

**Figure 2:**
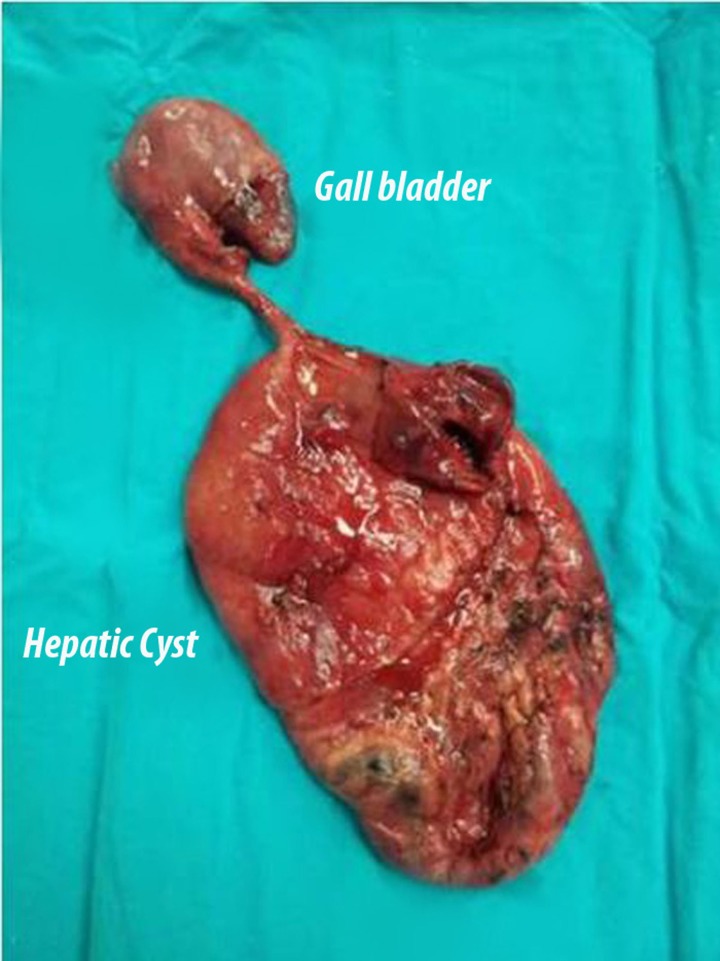
Postoperative image of the giant cyst.

Due to the patient’s age, the size of the cyst and cardiopulmonary failure, open surgery was performed. During abdominal exploration, a gaint SC originating from the gallbladder extendeding to the pelvic inlet, pushing gallbladder and the intestines downwards was detected. There was no pathological changes in other abdominal organs. Cystectomy and cholecystectomy was performed (Fig. 2). Pathologically benign SC of the liver and chronic cholecystitis was reported. With no mortality and morbidity, the blood sample taken 3 months after the surgery showed decreased CA 19-9, CA 15-3 and CEA levels of 36 IU/L, 28.1 IU/L and 3.05 ng/mL, respectively.

## Discussion

Although liver SC is usually asymptomatic, 10-15% of the cases can be symptomatic [1]. Liver SC is often diagnosed incidentally during abdominal surgery or imaging. Single or multiple cysts vary from a few millimeters to about 20 cm in size [3]. As the size of the cysts increases the frequency of being symptomatic also increases. In this case, the liver SC was giant in size, unilocular and solitary.

Diagnosis of the liver SC is generally made by imaging methods starting with USG. CT does not yield any more information than USG and magnetic resonance imaging (MRI). CT can be helpful in differetiating other abnormalities [4]. In the presented case, CT was performed in order to rule out any accompanying abnormalities.

Five percent of the patients may develop complications. Bleeding inside the cyst and infection are the most common complications. Less frequently, traumatic or spontaneous rupture, torsion, pressure on surrounding tissues (vena cava inferior compression results in lower extremity edema, portal vein compression results in portal hypertension,while pressure on the biliary tract results in cholangitis or jaundice), duodenum or bile ducts fistula may be seen [1].

Asymptomatic liver SCs are generally followed without the need for treatment. Aspiration-sclerotherapy may be preferred in symptomatic patients [5]. Because aspiration of the cyst alone is not enough, it should not be prefered due to risk of infection and high recurrence rate [1].

In recent years, minimally invasive surgery is preferred in the removal of SC of the liver as in many other operative approaches. However, laparoscopic fenestration carries a 10-25% risk of recurrence and 8.3% risk of conversion from close to open surgical procedure. In some cases, the morbidity rate reaches 33%. Therefore, it is recommended that laparoscopic fenestration should be used in easily accessible areas, superficial cysts especially located in the anteriorly or laterally of the abdomen, and that the traditional open surgery should be used in potential malignant cases where the cysts are posterior or are located near big vascular structures [3].

SCs of the liver are benign lesions that do not have any connection with intrahepatic biliary system. SCs can produce significant amount of liquid and biological markers such as CA 19-9 and CEA [6]. In many research studies it has been shown that the CA 19-9 values increase in the serum and in the cystic fluid. CA 19-9 may show increase in benign liver disease in addition to its use as a biomarker in differentiating between benign and malignant gastrointestinal disease [7]. Yoshida et al [8] presented article on infected hepatic liver cyst where high CA 19-9 values were observed. Similarly Ogawa et al [9] and Sawabu et al [10] discovered increases in CA 19-9 levels due to inflammatory pseudotumor of the liver and complicated cholelithiasis respectively. None of these abonormalities were seen in the case presented here. The authors believe that high values of CA 19-9 and CA 15-3 may be probably due to the pressure on the surrounding organs without any clinical symptoms, and may aslo be with a high probability due to excessive secretion from the cystic epithelialium due to the width of the inner face of the cyst. CA 19-9 and CA 15-3 values and even the height of creating clinical symptoms, yet with low probability have to press the surrounding organs, more likely, depending on the width of the inner face of the cyst epithelial cysts believe that is due to the excessive secretion. Although the high levels of preoperative CEA may be seen as a non-supportive aspect of the proposed hypothesis, the decreasing value of CEA during the postoperative period supports the hypothesis.

The authors believe that there is a need for comprehensive studies to investigate the relationship between the size cyst and biomarkers (including markers such as CA 15-3) in the assesment of liver SC.
